# Infrared Thermography of the Blowhole as a Potential Diagnostic Tool for Health Assessment in Killer Whales (*Orcinus orca*)

**DOI:** 10.3390/ani14131867

**Published:** 2024-06-25

**Authors:** Jennifer P. Russell, Steve D. Osborn, Kelsey E. S. Herrick, Todd L. Schmitt, Todd Robeck

**Affiliations:** 1 SeaWorld San Diego Zoological Department, 500 SeaWorld Drive, San Diego, CA 92109, USA; 2 SeaWorld San Antonio Zoological Department, 10500 SeaWorld Drive, San Antonio, TX 78251, USA; 3 SeaWorld Orlando Zoological Department, 7007 SeaWorld Drive, Orlando, FL 32821, USA; 4 SeaWorld and Busch Gardens Species Preservation Laboratory, 2595 Ingraham St., San Diego, CA 92109, USA

**Keywords:** infrared thermography, blowhole, killer whale, *Orcinus orca*

## Abstract

**Simple Summary:**

Non-invasive techniques, such as infrared thermography, are ideal diagnostic tools for evaluation of the health status of marine megafauna such as killer whales (*Orcinus orca*). Infrared thermography of the blowhole was implemented in the diagnostic work-up of two male killer whales (also known as ‘orcas’) under managed care, and accurately detected changes in blowhole mucosa emissivity consistent with pyrexia in both cases. Infrared thermography of the blowhole is a potentially useful addition for the health assessment of killer whales as a screening tool to identify pyrexic animals within a group. The aim of this case report is to describe the clinical presentation and diagnostic findings in two killer whales with signs consistent with inflammation and pyrexia, most notably that an increase in blowhole temperature obtained by infrared thermography aligned with increased rectal temperature.

**Abstract:**

Killer whales (*Orcinus orca*) are experiencing increasing environmental pressures, with some ecotypes being identified as endangered, and the development and validation of non-invasive health assessment tools is critical for assessing the well-being of individuals within these endangered populations. Infrared thermography of the blowhole is a non-contact method of temperature measurement that was recently investigated in killer whales in managed care. Two male killer whales presenting with clinical signs at separate institutions had veterinary clinical health assessments performed, which included infrared thermography of the blowhole as well as concurrent rectal temperature measurement. The current case report is aimed at describing the clinical use of infrared thermography of the blowhole as a method to detect elevated body temperature in two killer whales. Both animals exhibited blowhole temperatures above the previously reported values (36.4 °C and 37.6 °C; the mean in healthy whales is reported to be 34.21 ± 1.47 °C) with concurrently elevated rectal temperatures, as well as clinicopathologic findings consistent with a systemic inflammatory response (e.g., neutrophilia, increased fibrinogen and erythrocyte sedimentation rate, hypoferritinemia). Following veterinary intervention, both animals’ blowhole and rectal temperatures returned to baseline. Infrared thermography of the blowhole represents a promising tool for the identification of pyrexic animals and with further investigation may be considered as part of conservation health assessments for threatened free-ranging populations.

## 1. Introduction

As apex predators, killer whales (*Orcinus orca*) accumulate legacy chemical pollutants, such as polychlorinated biphenyl (PCBs) and mercury, and are one of the most contaminated marine mammals in the world [1,2,3]. While prey availability and anthropogenic factors are among the largest threats to wild populations, infectious disease is also a commonly reported cause of death, possibly due to the immunosuppressive effects of contaminants, malnutrition, or physiologic stress imposed by anthropogenic factors [1,4,5]. The validation of non-invasive methods for diagnostics and data collection is important due to the logistic difficulty of collecting direct physiologic samples or measurements from aquatic megafauna [6,7]. For animals that may be experiencing disease processes, non-invasive techniques are ideal. Stress associated with handling may compromise the individual further by disrupting social interactions, appetite, or willingness to participate in wellness exams [8,9]. Stress associated with handling naive or wild cetaceans can potentially result in stress-induced hyperthermia, electrolyte derangements, changes in circulating thyroid and adrenal hormones, or exertional rhabdomyolysis [9,10,11,12,13]. Non-invasive methods also circumvent any complications associated with the use of chemical restraint [14].

Health assessment of marine megafauna, such as killer whales, presents unique challenges to the veterinarian. These animals are often too large for traditional diagnostic imaging modalities (e.g., ultrasound tissue penetration is non-diagnostic at depths greater than 30 cm with commercially available equipment, and, similarly, radiographic imaging is limited by animal size and beam penetration [14,15,16]). Moreover, they live exclusively in an aquatic environment, where simple physical examination can be logistically difficult, and even something as simple as thoracic auscultation is inhibited by a thick blubber layer, rapid breath cycle, and water interfering with equipment [15]. This is in addition to the major constraint posed by unwell animals who may become non-participatory due to feelings of malaise, or free-ranging animals who are not directly accessible. Rectal temperature is used as part of the routine veterinary exam; however, this data can only be regularly collected if the animal is conditioned to accept a rectal probe and willing to participate. Until recently, no reference ranges for rectal temperature in killer whales were available [17].

Core body temperature is a physiological parameter that provides insight into clinical health status, and estimation of core body temperature (typically via rectal temperature measurement) is part of the minimum diagnostic database in veterinary medicine [18,19]. Infrared thermography can detect anatomical regions in many species that may serve as non-invasive proxies to core body temperature, known as thermal windows [20,21]. These are body surfaces that exhibit elevated heat exchange from the animal to the environment, and they typically have quantitative and qualitative differences in vascularization as compared to well-insulated areas of the body [21]. By identifying and using appropriate thermal windows, infrared imaging has the potential to recognize vasomotor changes consistent with disease processes that induce inflammation or pyrexia [22,23].

The blowhole of free-ranging and managed care cetaceans has been shown to provide a consistently detectable heat anomaly, given the lack of insulating blubber and large venous plexuses associated with the accessory sinus system [17,24,25,26,27]. This creates a potentially useful thermal window for infrared imaging. Infrared thermography of the blowhole was found to be nearly identical to rectal temperatures collected from bottlenose dolphins (*Tursiops truncatus*) and approximately 1 °C lower than rectal temperatures in beluga whales (*Delphinapterus leucas*) and 1.28 °C lower than rectal temperatures in killer whales [17,26]. Recent data comparing infrared thermography of the blowhole to rectal temperature in killer whales showed that the accuracy of the infrared blowhole reading improved as the mean temperature of the animal increased (i.e., as the mean rectal and blowhole temperatures increased, so did the accuracy of the infrared camera) [17]. Thus, it is possible that infrared imaging of the blowhole may have utility as a non-invasive diagnostic screening tool to identify pyrexic individuals in a population of whales.

A reliable non-contact method of evaluating body temperature, such as infrared thermography, may not only have implications for individual animals receiving veterinary care, but may also serve as a useful data collection tool when performing health assessments of wild killer whale populations [7,18,28,29]. Studies using infrared thermography in marine mammals are limited, and, to the authors’ knowledge, there are no case reports describing the use of infrared thermography of the blowhole in killer whales as part of a clinical diagnostic work-up. It is the authors’ objective to retrospectively report the clinical use of infrared thermography of the blowhole in two killer whale cases and discuss how detectable changes in blowhole temperature complement the assessment of health. The normality of clinicopathological results (i.e., hematology, biochemistry, and rectal and blowhole temperatures) was determined by comparisons with our previously reported killer whale age- and sex-specific reference intervals (RIs) [17,30,31], as well as comparisons with historical individual baseline data. Any value outside of these ranges was considered abnormal.

## 2. Case 1

**Figure 1:**
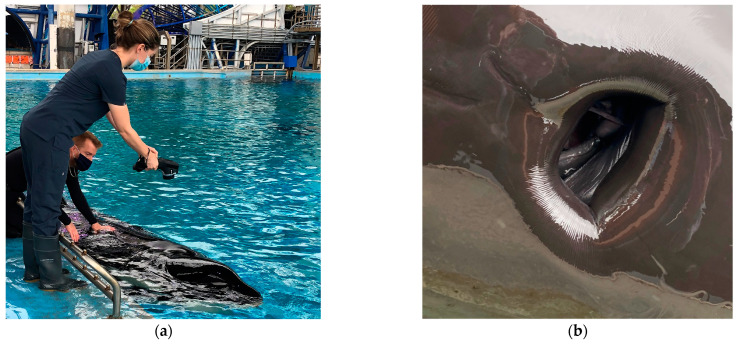
(**a**) A veterinarian performing an infrared blowhole temperature reading in a killer whale resting at the surface of the water. (**b**) A digital photograph of the open blowhole of a killer whale, for reference.

**Figure 2:**
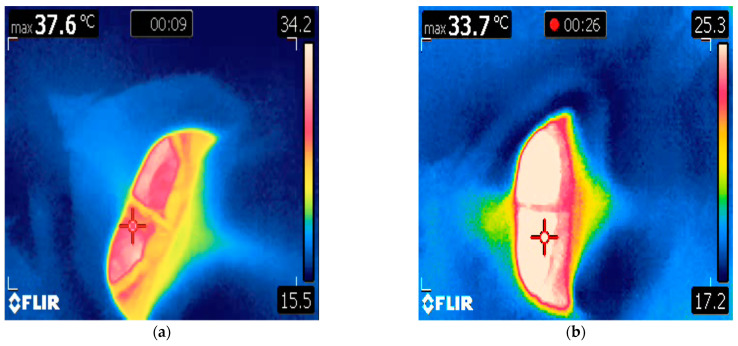
(**a**) A still image from an infrared video clip of the open blowhole of a male killer whale (*Orcinus orca*) described in Case 1 on the day of presentation. The red crosshair, or ‘hotspot’, tracks the maximum temperature within the image frame and reports it in the top left corner (37.6 °C). The bar on the right depicts the range of pixel colors assigned to temperature values. (**b**) A still image from an infrared video clip of the blowhole of the same killer whale following veterinary intervention.

A 10-year-old male killer whale living at Facility A, weighing 2561 kg, presented with slow movement through the habitat, halitosis, and regurgitation of food items. Appetite and general demeanor toward animal care staff were unchanged and husbandry behaviors were offered by the animal voluntarily. Veterinary physical examination and infrared thermography were performed on the same day as clinical signs were first reported. Infrared thermography of the oral mucosa was unremarkable. The whale was asked by husbandry staff to rest in ventral recumbency at the surface of the water and breathe normally while a handheld infrared camera (FLIR E40 Thermal Imaging Camera, Teledyne FLIR, 2700 SW Parkway Ave., Wilsonville, OR 97070, USA) was held directly above the blowhole at a distance of approximately 2 ft (0.61 m), as previously described (Figure 1a) [17]. The camera was calibrated prior to the exam using melting ice at 0 °C. An infrared video was taken of the blowhole opening and closing during a natural breath cycle, while a dynamic crosshair tracked the hottest area visible within the image frame. Blowhole temperature was 37.6 °C (Figure 2a). The reported mean observed blowhole range for healthy killer whales is 34.21 ± 1.47 °C [17]. The animal was then asked by trainers to rest in dorsal recumbency for insertion of a rectal temperature probe to 40 cm depth (Digi-Sense Temp Series Thermocouple thermometer, Cole-Palmer, 625 East Bunker Court, Vernon Hills, LI 60061, USA). Rectal temperature at presentation was 36.8 °C (the expected temperature range for male killer whales <4000 kg [ETR] is 35.10 to 35.50 °C [17]). A blood sample from the ventral fluke periarterial vascular rete (PAVR) revealed a decreased alkaline phosphatase (ALP) (497 IU/L, down from 531 IU/L), elevated erythrocyte sedimentation rate (ESR) (5 mm/h; 0 mm/h is typical for the species), increased fibrinogen (441 mg/dL, up from 227 mg/dL; RI 250.6 to 281.8 mg/dL), increased globulin (3.27 g/dL, up from 2.88 g/dL; RI 2.54 to 2.92 g/dL), decreased serum iron (26 µg/dL, down from 86 µg/dL; RI 69.3 to 86.6 µg/dL), and increased white blood cell count (WBC) (6.60 × 10^3^/µL with 82% relative neutrophilia, up from 5.13 × 10^3^/µL; RI 5.32–6.07 × 10^3^/µL). These changes are consistent with a systemic inflammatory response and were notably changed from a routine sample collected two weeks prior to presentation. The animal was started on empiric treatment with levofloxacin (5 mg/kg orally [PO] q 24 h), amoxicillin clavulanate (7 mg/kg PO q 12 h), voriconazole (0.27 mg/kg PO q 24 h), and famotidine (0.4 mg/kg PO q 24 h) [32]. Diagnostic imaging was performed, including voluntary dental radiographs (unremarkable) and abdominal and thoracic ultrasound (unremarkable except for a subjective decrease in intestinal motility from baseline). Ancillary diagnostic investigation included blowhole sputum sampling (for anaerobic, aerobic, fungal, and acid-fast culture and sensitivity and respiratory microbiome characterization [via next generation DNA sequencing] and avian influenza polymerase chain reaction [PCR] [negative]) as well as whole blood pan-bacterial and pan-fungal PCR (negative), fecal cytology and gram stain (unremarkable), enteric viral PCR (negative), and fecal microbiome characterization. Findings were supportive of a presumptive diagnosis of uncomplicated gastroenteritis, which responded well to continued supportive treatment (famotidine was discontinued, and omeprazole [360 mg total dose PO q 24 h] and sucralfate [20 g total dose PO q 8 h] were initiated [32]). Rectal temperature decreased back to baseline to 35.5 °C, and blowhole temperature was 33.7 °C three days after initiating treatment (Figure 2b). Full clinical resolution was achieved within 15 days of initial presentation with normalization of complete blood count (CBC), serum biochemistry, ESR, and fibrinogen, and medications were discontinued on day 18.

## 3. Case 2

**Figure 3:**
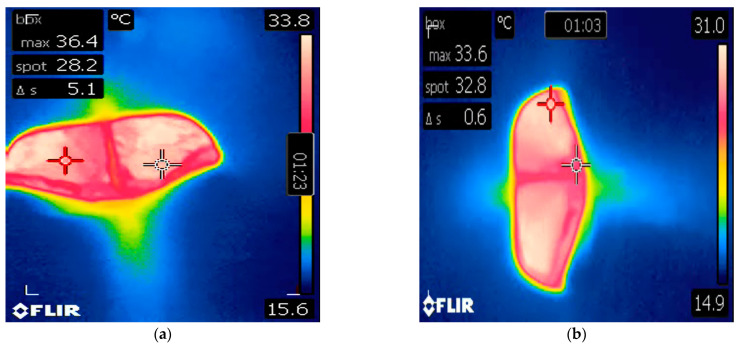
(**a**) A still image from an infrared video of the open blowhole of a killer whale (*Orcinus orca*) described in Case 2. The camera in this case was in the ‘Hotspot-Spot’ setting, whereby a red crosshair dynamically tracks the hottest detectable pixel within the frame (‘max’) and a white crosshair remains static in the center of the screen (‘spot’). The blowhole temperature in this case was recorded as 36.4 °C. (**b**) A still image from an infrared video clip of the blowhole of the same killer whale following veterinary intervention.

A 21-year-old male killer whale living at Facility B, weighing 3672 kg, presented with decreased appetite and resting at the bottom of the pool. Veterinary physical exam was performed on the same day as clinical signs were reported. Infrared thermography of the oral mucosa revealed an increase in surface temperature at the lower right eighth tooth, which had associated periapical lucency on voluntary dental radiographs. The animal was asked by husbandry staff to rest in ventral recumbency at the surface of the water and breathe normally while a handheld infrared camera (the same model as in Facility A) was held directly above the blowhole at a distance of approximately 2 ft (0.61 m), as previously described [17]. The camera was calibrated using melting ice at 0 °C prior to examination. Blowhole temperature was 36.4 °C on the day of presentation (Figure 3a). The animal was then asked by trainers to rest by the side of the pool in dorsal recumbency for insertion of a rectal temperature probe to 40 cm depth. Rectal temperature was 37.4 °C. A blood sample collected the same day from the ventral fluke PAVR revealed decreased ALP (69 IU/L, down from 100 IU/L), markedly elevated ESR (23 mm/h; 0 mm/h is typical for the species) and fibrinogen (608 mg/dL, up from 265 mg/dL), globulin (4.4 g/dL, up from 3.7 g/dL), decreased iron (23 µg/dL, down from 75 µg/dL), and leukocytosis (9.46 × 10^3^/µL characterized by an 86% neutrophilia, up from 6.08 × 10^3^/µL). These changes are consistent with a systemic inflammatory response, are outside of the available reference intervals for this species [17,30,31], and were notably changed from a routine sample collected one week prior to presentation. This animal was diagnosed with a periapical tooth abscess and a dental extraction was subsequently performed. The animal responded well to antimicrobial therapy and analgesia (clindamycin 4.5 mg/kg PO q 12 h, voriconazole 0.27 mg/kg PO q 24 h, and aspirin 240 grain equine bolus total dose PO once). Rectal and blowhole temperatures returned to 35.5 °C and 33.6 °C, respectively, within one week of initiating treatment (Figure 3b). Normalization of CBC, serum biochemistry, ESR, and fibrinogen followed dental extraction performed 14 days later.

## 4. Discussion

In both cases, blowhole and rectal temperatures simultaneously increased outside of the reported observed healthy range for the species [17] and, in the context of concurrent diagnostic results, were interpreted as pyrexia for these individuals. The additional information provided by the blood analyses and diagnostic imaging supported working diagnoses of inflammatory processes resulting in peripheral vasodilation and increased thermal radiation consistent with a pyrexic state. Rigorous monitoring of the blood parameters and temperatures of these individuals was important to document both a positive response to treatment and clinical resolution.

Infrared thermography is not a direct measurement of temperature but rather the detection of infrared radiation emitted by a subject. This is translated into an image, which depicts each infrared energy level using arbitrarily assigned colors [21,33]. In these cases, infrared blowhole temperature measurement and rectal temperature via rectal probe were used as surrogates of core body temperature, as invasive absolute core body temperature measurement was not performed. In each case, different disease processes resulted in a similar systemic inflammatory response. In mammals, the release of pyrogens and proinflammatory cytokines alter the thermal ‘set point’ in the anterior hypothalamus and cause an increase in core body temperature. This, in turn, results in changes in peripheral vasodilation and heat exchange with the environment [21,22,34,35,36]. Pyrexia is distinct from nonfebrile hyperthermia, where the hypothalamus is not influenced, but is rather a function of heat gain vs. loss (e.g., during exercise, inadequate heat dissipation, etc.) [34,35,37]. This is a key concept to note if performing infrared thermography on animals where physical or chemical restraint is employed, as stress-induced hyperthermia may occur [8,9].

While case number is a limitation in the current report, infrared thermography of the blowhole as a complimentary diagnostic tool proved to be reliable at detecting systemic inflammation and pyrexia. It was well-tolerated and comfortable for the animals (who readily participated), was safe to perform for both animal and clinician, and provided informative insight into the thermoregulatory changes associated with the clinical state of the animals, allowing for timely and successful treatment to be initiated. In Case 2, the use of aspirin as an analgesic may have contributed to the reduction in rectal and blowhole temperature as it is also an antipyretic agent. Nevertheless, infrared thermography was able to detect this reduction in temperature.

Infrared temperature measurement of the blowhole was performed in these cases in a clinical setting and not under controlled experimental conditions. While these animals were participatory and conditioned to rest at the surface for blowhole and rectal temperature measurement, infrared thermography is a non-contact method that may theoretically be used in individuals that are not conditioned, non-participatory due to malaise or other factors, or not amenable to handling, such as in the case of free-ranging animals. Such assessments may potentially be incorporated into data collection performed via unmanned aerial systems (drones) as previously described [7,24,38,39,40,41]; however, further investigation is required, given that there are multiple factors that may influence the infrared temperature measurement of the blowhole of a free-ranging animal, including altitude or distance from the subject, air temperature, water temperature, wind speed, cloud cover, emittance of surrounding sea water, the animals’ wet skin surface, or blowhole vapor interference [17,21,22,38,42]. It is also unknown whether blowhole temperatures will vary beyond what is observed here during swimming or exercise. Further investigation is required to determine if killer whales become hyperthermic with exercise and whether this is detectable via infrared imaging. In studies examining the rectal temperature of bottlenose dolphins, it was found that rectal temperatures obtained during exercise were 0.5 °C cooler than temperatures measured at rest [43].

Additional case report data are required to investigate whether infrared thermography of the blowhole can accurately detect clinically significant temperature elevation in various disease states beyond those described here, or whether it might be a useful measurement tool to detect decreases in core body temperature associated with parturition in females [44,45]. The two individual case examples reported herein may provide evidence to support the use of blowhole thermography for identifying pyrexia in animals resting at the surface of the water. However, it cannot be assumed that a systemically unwell animal will always have a blowhole temperature above the normal range. Given that the tendency for blowhole temperature readings is to be below those of rectal temperature in the healthy animal, and that accuracy improves with increasing mean temperature, it is unlikely that this temperature measurement will yield false positives; however, more clinical case data are needed to investigate this [17]. In Case 1, the blowhole temperature recorded was higher than the rectal temperature, which supports this concept. While rectal temperature is now performed daily as part of the routine clinical health assessment for killer whales in the authors’ facilities, the utility of blowhole temperature has only recently been investigated in this species [17]. Only two clinical cases incorporating infrared temperature measurement of the blowhole have occurred within the last three years, and these are the only two animals to date that have had measured blowhole temperatures above the observed ‘normal’ range [17]. 

## 5. Conclusions

Infrared thermography of the blowhole was a reliable sensor for inflammation and pyrexia in two killer whales presenting with clinical signs. This non-invasive method was well-tolerated by the animals with no adverse effects. Infrared thermography of the blowhole has clinical application for managed-care individual animals receiving veterinary care (particularly animals that are not amenable to rectal temperature measurement) and may possibly also serve as a useful screening tool when performing health assessments of free-ranging killer whale populations via unmanned aerial systems, allowing the detection of pyrexic animals within a group. Additional case report data incorporating blowhole infrared thermography as part of the diagnostic toolkit will help to corroborate these findings.

The detection of pyrexic animals might be beneficial for epidemiologists investigating infectious or inflammatory disease prevalence within a population (e.g., animals identified as pyrexic may provide insight for serological surveys or stranding data as to whether animals sampled are demonstrating seroconversion due to previous disease exposure vs. active infection). It may benefit the clinical evaluation of live stranded animals to assist rehabilitative efforts or the identification of individuals in need of veterinary or other intervention (e.g., via direct or remote delivery of therapeutics [46]). It might assist in the identification of immunocompromised individuals in a critically endangered population or in the investigation of the physiologic effects of various environmental or anthropogenic influences, which may provide valuable data in support of species protective legislation or other influential conservation measures. Blowhole temperature data from managed-care and free-ranging killer whales and other cetacean species may therefore provide valuable contextual data that will compliment assessments of health status for wild populations.

## Data Availability

No new data were created for this report.
